# Posterior Fossa Arachnoid Cyst Masking a Delayed Diagnosis of Hyperparathyroidism in a Child

**DOI:** 10.1155/2012/931371

**Published:** 2012-11-25

**Authors:** B. Dhamija, D. Kombogiorgas, I. Hussain, G. A. Solanki

**Affiliations:** Department of Neurosurgery, Birmingham Children's Hospital, Steelhouse Lane, Birmingham B4 6NH, UK

## Abstract

*Background*. Primary hyperparathyroidism in childhood is a very rare entity, often being diagnosed late after the onset of its presenting symptoms. It most commonly affects patients in their fourth decade of life and beyond. The inclusion of primary hyperparathyroidism in the differential diagnosis is necessary when evaluating patients presenting with nonspecific symptoms such as polyuria, fatigue, weight loss, abdominal pain, nausea, and vomiting. *Methods*. We report the case of an eleven-year-old girl presenting with three years history of headaches, visual disturbance, along with episodes of emotional lability. Neuroimaging confirmed a large posterior fossa arachnoid cyst. It was decided to manage this lesion conservatively with surveillance. Only after further hospital admissions with recurrent loss of consciousness, dizziness, and nausea to add to her already existing symptoms, a full biochemical and endocrine assessment was performed to look for more specific causes for her presentation. These pointed to a diagnosis of primary hyperparathyroidism. *Conclusions*. The inclusion of primary hyperparathyroidism in the differential diagnosis should be considered when evaluating paediatric patients presenting with nonspecific (neurological, gastrointestinal, and renal) symptoms in order to establish a prompt diagnosis of the disorder and to avoid severe complications of prolonged hypercalcaemia and end-organ damage.

## 1. Introduction

Primary hyperparathyroidism is a well-described condition in adults with an annual incidence between 25 and 50 per 100,000 general population [1, 2]. It most commonly affects patients in their fourth decade of life and beyond [1]. However, primary hyperparathyroidism in childhood is an extremely rare entity, which very often is diagnosed with significant delay following the commencement of its presenting symptoms [3–6]. Inclusion of primary hyperparathyroidism in the differential diagnosis is required when evaluating paediatric patients, who present with nonspecific symptoms which include polyuria, fatigue, weight loss, abdominal pain, nausea, and vomiting. Prompt diagnosis of primary hyperparathyroidism can avoid severe complications of prolonged hypercalcaemia and end-organ damage such as nephrocalcinosis, nephrolithiasis, bone demineralization, and mental retardation [3, 4]. We report the case of an eleven years old girl with three years history of headaches, visual disturbance, episodes of emotional lability, and a posterior fossa large arachnoid cyst. Despite her repeated admissions to hospital, the incidental finding of a posterior fossa arachnoid cyst distracted clinicians resulting in delayed diagnosis of her primary hyperparathyroidism.

## 2. Case Presentation

We report an 11-year-old girl with a known history of febrile convulsions during the first year of life and coeliac disease diagnosed at the age 8 years, presenting with a one-year history of intermittent nocturnal and early morning headaches and horizontal diplopia. Neurosurgical referral followed from a provisional diagnosis of brain space occupying lesion. 

Initial neurological examination revealed a mild left sided dysmetria and minor trunk ataxia. Ophthalmological examination did not reveal any specific abnormality, there was no extraocular muscle dysfunction or gaze paresis. 

**Figure 1:**
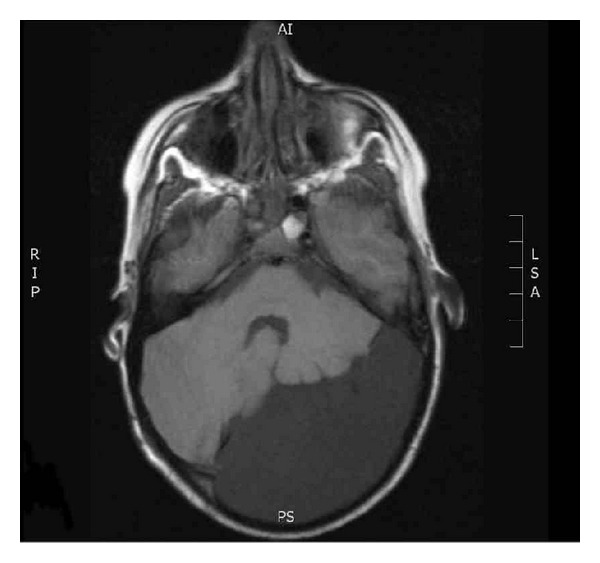
Axial MRI Head scans showing the presence of a large posterior fossa arachnoid cyst, this is predominantly left sided, causing mild midline shift and some distortion of the ventricular system. There is no hydrocephalus.

**Figure 2:**
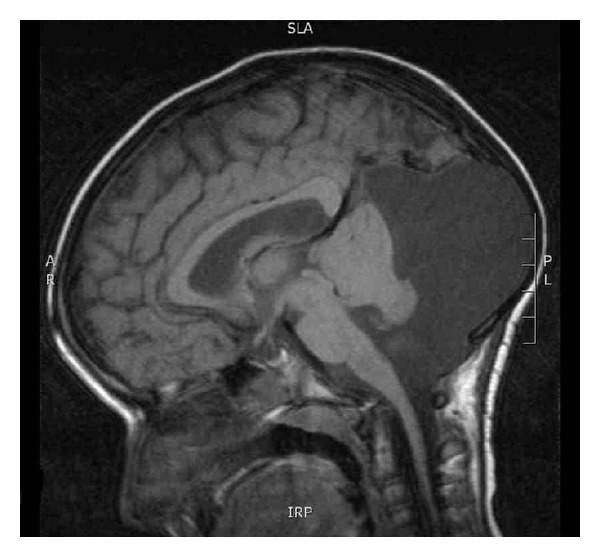
Sagittal MRI Head scans showing the presence of a large posterior fossa arachnoid cyst, this is predominantly left sided, causing mild midline shift and some distortion of the ventricular system. There is no hydrocephalus.

CT and MRI imaging of the brain showed a left infratentorial arachnoid cyst causing mild compression on the cerebellum and scalloping of the occipital skull vault. There was no evidence of hydrocephalus (Figures 1 and 2). These findings were not sufficient to explain all her presenting symptoms and conservative management of the arachnoid cyst was agreed. 

A few months later, she was readmitted to hospital with episodes of recurrent loss of consciousness, visual disturbances, and dizziness. These were preceded by nausea and headaches. She had no loss of continence with these episodes, which could last up to an hour. She showed a rapid beating nystagmus but was responsive and able to talk during these episodes. She appeared tearful and tired afterwards. She had no focal neurological signs and no evidence of raised intracranial pressure. Of note, she had no cerebellar signs. These episodes occurred particularly in the morning, and she became unable to walk unaided. She suffered from anxiety and emotional lability and was unhappy at school. EEG performed during one of these episodes of emotional lability was normal. A psychiatric assessment established the diagnosis of emotional lability attacks rather than seizures. Subsequently, haematological, biochemical, and endocrinological assessment was performed.

Full blood count was normal. Venous blood sampling revealed adjusted calcium of 2.83 mmol/L (normal range: 2.20–2.60 mmol/L), phosphate of 0.96 mmol/L (normal range: 1.20–1.80 mmol/L), and parathyroid hormone of 83 ng/L (normal range: 13–29 ng/L). Serum sodium, potassium, urea, creatinine, magnesium, liver function, and thyroid function tests were normal. Urine calcium/creatinine ratio was 0.76 mmol/mmol (normal range: 0.00–0.70 mmol/mmol). Serial venous blood sampling showed persistently high calcium levels of 2.89 mmol/L and 2.76 mmol/L. A repeat PTH remained high at 91 ng/L. The mother's venous blood adjusted calcium was normal (2.37 mmol/L, normal range: 2.20–2.60 mmol/L), excluding the possibility of benign familial hypercalcaemia. Consequently, a diagnosis of primary hyperparathyroidism was made.

A renal ultrasound investigation showed structurally normal kidneys with no evidence of calculi. X-rays of the patient's hands did not show subperiosteal erosions of the distal phalanges. A parathyroid isotope scan did not identify any abnormal parathyroid gland uptake. 

Our patient underwent psychiatric therapy support and medical management of her hyperparathyroidism. Subsequent one year followup showed she was making satisfactory progress and had good control of her hypercalcaemia.

## 3. Discussion 

Primary hyperparathyroidism occurs due to an excessive and inappropriate secretion of PTH, the hallmark of the disorder is hypercalcaemia. In contrast, secondary hyperparathyroidism is characterized by hypocalcaemia leading to a compensatory increase in secretion of PTH [7]. 

Physicians frequently fail to check serum calcium and parathyroid hormone levels when evaluating children with nonspecific complaints such as polyuria, fatigue, weight loss, abdominal pain, nausea, and vomiting [3, 4] (Table 1).

Primary hyperparathyroidism in childhood is a very rare entity, it is very often diagnosed with significant delay after the commencement of its presenting symptoms [3–6]. Kollars et al. reported a series of 52 patients under the age of 19 years at the time of diagnosis. 41 (79%) of 52 patients were symptomatic at presentation. In 19 patients with a known specific time of symptom onset, the median time until diagnosis was 24 months (range: 1–60 months) [4]. Only 3 (5.77%) of these 52 patients were under the age of 10 years at the time of diagnosis [4]. Most patients diagnosed with primary hyperparathyroidism are older than 16 years of age [4, 8]. It is slightly more common in females than males with a female-to-male ratio 3 : 2 [4, 8, 9]. The incidence of disease in infancy is exceedingly rare [10]. 

Primary hyperparathyroidism may be sporadic or familial. Sporadic hyperparathyroidism in older children is nearly always due to a single parathyroid adenoma, and the gender incidence is equal [7]. Familial hyperparathyroidism is usually caused by chief cell hyperplasia of all four parathyroid glands. It is important to distinguish this from familial benign hypercalcemia (familial hypocalciuric hypercalcemia) [11], because the latter is a truly benign condition, and parathyroid surgery should not be performed [7]. Primary hyperparathyroidism may occur in association with multiple endocrine neoplasia type 1 or 2 (MEN-1, MEN-2) [7]. Neonatal primary hyperparathyroidism is a severe and often life-threatening disorder. These infants typically display severe hypercalcemia (3.75–7.5 mmol/L), respiratory distress, muscular hypotonia, and skeletal demineralization. They are usually diagnosed within the first three months of life and have hyperplasia of all four parathyroid glands [7, 10]. 

In older children primary hyperparathyroidism can be asymptomatic or may have subtle, nonspecific symptomatology including, fatigue, headache, abdominal pain, nausea, vomiting, poor appetite, weight loss, polydipsia, polyuria, haematuria, diarrhoea, constipation, depression, joint pain, back/bone pain, bone deformities, irritability, and insomnia [3, 4, 8, 9]. Thoracic deformities also predispose to recurrent pneumonia [3]. Cardiac abnormalities have been reported in patients with high levels of PTH, which may lead to circulatory problems and even death [12] and can also contribute to the high frequency of pneumonia [3].

End-organ damage is frequently recognised at the time of presentation of children with primary hyperparathyroidism. It includes nephrolithiasis (33%–54%) [4, 8, 13], bone disease (27%–34%) [4, 8, 13], nephrocalcinosis (8%–70%) [3, 4], and pancreatitis (3%–7%) [4, 13]. The broad category of end-organ damage is not frequent in younger patients [4]. 

The extracellular calcium-sensing receptor (CaSR) plays a key role in calcium homeostasis, as it serves as the body's “thermostat” for calcium [14]. The CaSR gene is located on chromosome 3q21.1. The CaSR contributes to maintaining normal physiological levels of serum calcium concentration by inhibiting PTH secretion and promoting renal calcium excretion in response to an increase in serum calcium [14]. 

In severe neonatal hyperparathyroidism, there is homozygous mutations in the CaSR gene in children born to consanguineous familial hypocalciuric hypercalcemia (FHH) parents [15]. The degree of hypercalcaemia appears to reflect a gene dose effect [16]. Because these infants lack any normal copies of the CaSR gene, they exhibit severe resistance of the CaSR-expressing tissues, especially the parathyroid glands, to Cao^2+^ [17]. In contrast, in primary hyperparathyroidism there is an acquired tissue selective resistance of pathological parathyroid gland(s) to Cao^2+^. Consequently, there is an increase in the set point of one or more pathological parathyroid glands to Cao^2+^, thereby resetting the level of Cao^2+^ upward. The molecular basis for the development of the reduced CaSR expression that likely causes the decreased responsiveness of pathological parathyroid glands to Cao^2+^ in primary hyperparathyroidism is still not fully understood. 

As the diagnosis of primary hyperparathyroidism in paediatric patients is frequently delayed and has significant morbidity, clinicians need a high index of suspicion for hyperparathyroidism and a low threshold for carrying out relevant investigations. For children in whom primary hyperparathyroidism is suspected, evaluation of serum calcium and PTH levels are the diagnostic tests of choice [4]. 

The diagnosis of hyperparathyroidism rests mainly on the repeated finding of increased plasma concentrations of calcium (2.75–7.5 mmol/L). The severity of hypercalcemia depends on the type of parathyroid disorder and the age of the patient [7]. The serum phosphorus is reduced to about 1 mmol/L, and there is a tendency toward a hypomagnesemia [7]. Elevated alkaline phosphatase has mainly been found in children with adenoma and extensive bone lesions [7]. 

When an adenoma is identified, parathyroid resection is effective at restoring normal serum calcium and is the treatment of choice for children with primary hyperparathyroidism [4, 8]. Modalities of surgical intervention consist of cervical exploration with single-gland parathyroidectomy for isolated adenoma and subtotal parathyroidectomy or total parathyroidectomy with autotransplantation for multiple-gland disease or hyperplasia [4]. Postoperative complications include hypocalcemia (transient symptomatic hypocalcemia occurring in 46% of the patients) [4] and transient vocal cord paralysis (4%) [4, 8]. 

## 4. Conclusion

In this case, the presence of a space occupying lesion was not sufficient to warrant all of our patient's presenting symptoms. Our report shows that the inclusion of primary hyperparathyroidism in the differential diagnosis, even in the presence of other pathology, is warranted when evaluating paediatric patients, presenting with nonspecific (neurological, gastrointestinal, and renal) symptoms in order to establish a prompt diagnosis of the disorder and to avoid severe complications of prolonged hypercalcaemia and end-organ damage. 

## Figures and Tables

**Table 1:** Common symptoms and signs associated with hyperparathyroidism.

Renal	Abdominal	Orthopaedic	Neurological
Increased thirst leading to increased urination	Nausea, vomiting	Muscle weakness and fatigue	Depression
Kidney stones	Constipation	Generalised aches and pains	Confusion
	Loss of appetite	Increased propensity to develop fractures	Impairment of thinking and memory
	Upper abdominal pain	Decreased height	Personality changes
			Stupor, possibly coma

## Abbreviations

PHPT: Primary hyperparathyroidism
PTH: Parathyroid hormone
CT: Computerized tomography
MRI: Magnetic resonance imaging
Cao^2+^: Extracellular calcium concentration
CaSR: Cao^2+^-sensing receptor.
